# Advanced Deep Learning Models for Classifying Dental Diseases from Panoramic Radiographs

**DOI:** 10.3390/diagnostics16030503

**Published:** 2026-02-06

**Authors:** Deema M. Alnasser, Reema M. Alnasser, Wareef M. Alolayan, Shihanah S. Albadi, Haifa F. Alhasson, Amani A. Alkhamees, Shuaa S. Alharbi

**Affiliations:** 1 Department of Information Technology, College of Computer, Qassim University, Buraydah 52571, Saudi Arabia; 421202234@qu.edu.sa (D.M.A.); 421202130@qu.edu.sa (R.M.A.); 421202222@qu.edu.sa (W.M.A.); 421202205@qu.edu.sa (S.S.A.); hhson@qu.edu.sa (H.F.A.); 2 Department of Orthodontics and Pediatrics Dentistry, College of Dentistry, Qassim University, Buraydah 52571, Saudi Arabia; ama.alkhamees@qu.edu.sa

**Keywords:** artificial intelligence, dental diseases, deep learning methods, neural network, medical imaging, image classification

## Abstract

**Background/Objectives:** Dental diseases represent a great problem for oral health care, and early diagnosis is essential to reduce the risk of complications. Panoramic radiographs provide a detailed perspective of dental structures that is suitable for automated diagnostic methods. This paper aims to investigate the use of an advanced deep learning (DL) model for the multiclass classification of diseases at the sub-diagnosis level using panoramic radiographs to resolve the inconsistencies and skewed classes in the dataset. **Methods:** To classify and test the models, rich data of 10,580 high-quality panoramic radiographs, initially annotated in 93 classes and subsequently improved to 35 consolidated classes, was used. We applied extensive preprocessing techniques like class consolidation, mislabeled entry correction, redundancy removal and augmentation to reduce the ratio of class imbalance from 2560:1 to 61:1. Five modern convolutional neural network (CNN) architectures—InceptionV3, EfficientNetV2, DenseNet121, ResNet50, and VGG16—were assessed with respect to five metrics: accuracy, mean average precision (mAP), precision, recall, and F1-score. **Results:** InceptionV3 achieved the best performance with a 97.51% accuracy rate and a mAP of 96.61%, thus confirming its superior ability for diagnosing a wide range of dental conditions. The EfficientNetV2 and DenseNet121 models achieved accuracies of 97.04% and 96.70%, respectively, indicating strong classification performance. ResNet50 and VGG16 also yielded competitive accuracy values comparable to these models. **Conclusions:** Overall, the results show that deep learning models are successful in dental disease classification, especially the model with the highest accuracy, InceptionV3. New insights and clinical applications will be realized from a further study into dataset expansion, ensemble learning strategies, and the application of explainable artificial intelligence techniques. The findings provide a starting point for implementing automated diagnostic systems for dental diagnosis with greater efficiency, accuracy, and clinical utility in the deployment of oral healthcare.

## 1. Introduction

Dental diseases, such as cavities, fractured teeth, periapical lesions, impacted teeth, and bone loss, pose significant challenges for oral healthcare. The accurate detection and classification of these conditions are essential for effective treatment and prevention [1,2]. Panoramic radiographs provide a comprehensive view of the oral cavity, capturing the teeth, jaws, and surrounding structures in a single image, so they are invaluable for diagnosing multiple conditions simultaneously [3,4]. However, traditional diagnostic methods are time-intensive, prone to human error, and heavily reliant on clinician expertise, leading to inconsistencies and missed diagnoses, particularly for subtle or coexisting conditions [2,5].

Recent developments in artificial intelligence (AI), especially deep learning (DL), provide hopeful solutions to overcome these constraints [6]. Convolutional neural networks (CNNs), a key DL technique, have also shown a fantastic ability to process medical images and are being used for the automatic detection of dental conditions including caries, fractured teeth, and missing teeth [7]. CNNs can achieve high accuracy on limited annotated data by utilizing transfer learning [8]. AI systems in dental diagnostics have significantly improved diagnostic accuracy and efficiency, reducing human error and supporting dental professionals in providing timely and consistent care [2,9].

Despite these advancements, there are still concerns. These challenges are attributed to the low availability of high-quality annotated datasets [2,6], difficulty generalizing the models to a wider patient population [4], and the high implementation costs of the AI systems [2]. Furthermore, most of the available AI models are trained to predict a single condition, while patients frequently have more than one coexisting problem in dentistry [2,9]. However, a robust AI system is required to recognize multiple conditions concurrently using a single panoramic radiograph.

This study addresses these challenges by evaluating and comparing five state-of-the-art deep learning architectures—VGG16, ResNet50, DenseNet121, EfficientNetV2, and InceptionV3—for the multiclass classification of dental conditions using panoramic radiographs. By employing advanced preprocessing techniques and addressing class imbalance, this research aims to develop a robust and clinically applicable AI-based diagnostic tool to enhance diagnostic accuracy, improve patient outcomes, and streamline clinical workflows in dentistry.

## 2. Literature Review

Recent advancements in AI have driven innovative approaches across various domains, including dental disease detection. Numerous studies have explored the integration of AI techniques, such as machine learning and deep learning, to improve diagnostic accuracy, treatment planning, and overall efficiency in dental care. This section reviews key contributions, methodologies, and challenges in AI-driven dental diagnostics.

AI has been applied extensively in the diagnosis and classification of dental conditions. For instance, Kong et al. [10] developed a semantic segmentation model based on the ERFNet architecture using 2000 annotated panoramic radiographs across 15 dental-related classes. The model achieved high accuracy but encountered challenges with overlapping teeth. Mahdi et al. [11] employed Faster R-CNN with ResNet-50 and ResNet-101 backbones for tooth detection and numbering, achieving impressive mean average precision (mAP) values of 97.4% and 98.1%, respectively, though it lacked data augmentation and demographic diversity.

Kim et al. [12] introduced Faster R-CNN InceptionV3 for detecting teeth, implant fixtures, and crowns, achieving a 96.7% mAP for tooth detection. However, the accuracy for implant and crown detection remained lower due to variability in shapes. Similarly, Lin et al. [13] used ResNet-based CNN models to classify tooth positions and dental conditions, attaining accuracy rates of 95.62% and 98.33%, respectively.

Several studies have explored the performance of state-of-the-art deep learning architectures. Chen et al. [14] implemented Faster R-CNN Inception v2 to detect ten dental conditions, achieving an F1 score of 94.3% for implants. Almalki et al. [15] applied YOLOv3 to classify cavities, root canals, crowns, and broken-down roots, achieving remarkable accuracy of 99.33%. Muramatsu et al. [16] and Chauhan et al. [17] focused on CNNs for tooth classification and diagnosis. Muramatsu achieved 96.4% sensitivity for tooth detection, while Chauhan combined CNNs with fuzzy logic to diagnose pulpitis, achieving 94% accuracy, outperforming expert predictions. Further, Hasnain et al. [18] applied CNNs for binary classification of dental radiographs, reaching 97.87% accuracy. However, the binary nature of the study limited its applicability to complex dental conditions. Recent work by Hasnain et al. [19] explored EfficientNet for detecting cavities, fillings, and implants, with EfficientNet-B5 achieving an F1 score of 98.37% after addressing class imbalance using Borderline Synthetic Minority Oversampling Technique (BL-SMOTE).

To address the broader classification of dental conditions, Cejudo et al. [20] used ResNet-34 pretrained on ImageNet to classify multiple radiograph types, achieving over 97% accuracy. Al et al. [21] utilized NASNet with transfer learning to classify cavities, fillings, and implants, demonstrating promising accuracy. Rahman et al. [22] introduced a specialized dataset enhanced with Contrast Limited Adaptive Histogram Equalization (CLAHE) and You Only Look Once Version 7 (YOLOv7) based training for six dental categories, achieving a precision of 96.03% and recall of 86.53%.

While these studies demonstrate significant progress, there are some limitations, such as poor access to annotated datasets, population generalizability, and poor images quality. Models’ robustness is significantly improved through the integration of higher-level preprocessing techniques (e.g., CLAHE [23]), and ensemble methods (e.g., fuzzy logic [17]). Model building in the future should have the capacity to deal with simultaneous dental cases with scalable implementations and clinical utility.

Later, kuccuk et al. [24] proposed a hybrid CNN–transformer-based model that accurately detected impacted teeth on panoramic radiographs with a mAP of 98.3% and an F1 score of 96%, significantly improving diagnostic accuracy and efficiency in clinical dentistry. Zeng et al. [25] proposed a Multi-Scale Perception Gated Convolution-based YOLO (MMC-YOLO) framework combining multi-task learning with advanced feature fusion techniques, which enables the simultaneous detection and segmentation of multiple dental pathologies, achieving state-of-the-art performance on the Dental Panoramic Multi-Disease Dataset (DPMD). Deo et al. [26] integrated ResNet50 with DenseNet201 in an ensemble learning scheme based on a 2D empirical wavelet transform and achieved a classification accuracy of 92% for oral squamous cell carcinoma (OSCC) detection from histopathological images.

Recent studies have also emphasized the importance of validating the data augmentation strategies and tackle class imbalance in a step-wise way. For example, Onakpojeruo et al. [27] recently proposed a Pix2Pix generative adversarial network (GAN)-based augmentation framework for MRI brain tumor classification, demonstrating that realistic synthetic data generation can significantly mitigate class imbalance, with corresponding benefits for classification performance. They also underlined the significance of fine hyperparameter tuning and architectural choice in obtaining reliable performance under imbalanced medical imaging data. Their results are consistent with the approach taken in the methodology of this work, including class-specific augmentation and optimized deep learning model architectures for panoramic radiograph analysis.

Table 1 displays a wide spread of performance across various ML/DL architectures. Models such as ResNet-101 and YOLOv3 achieve high accuracy and precision in assessing dental pathologies, although many other models work better in different diagnostic targets. With different dataset sizes and types of radiography, generalizable results are urgently required and should be able to be efficiently transferred into a more heterogeneous clinical application.

Despite significant advancements in applying AI and DL to dental disease detection, several challenges remain. Key limitations include small and non-diverse datasets, which limit model generalizability to broader clinical settings, as highlighted by Turosz et al. [28] and Fukuda et al. [29]. Variability in image quality, particularly in panoramic radiographs and CBCT images, has been shown to negatively impact performance. Additionally, existing models struggle with early-stage disease detection and often focus on limited class categories, restricting their ability to address a wide range of dental conditions. Furthermore, reliance on private datasets limits accessibility and reproducibility, hindering model validation and improvement. Our study addresses these gaps by enhancing dataset diversity, improving model robustness, and expanding classification scope, aiming to develop a more clinically applicable AI solution.

## 3. Materials and Methods

**Figure 1:**
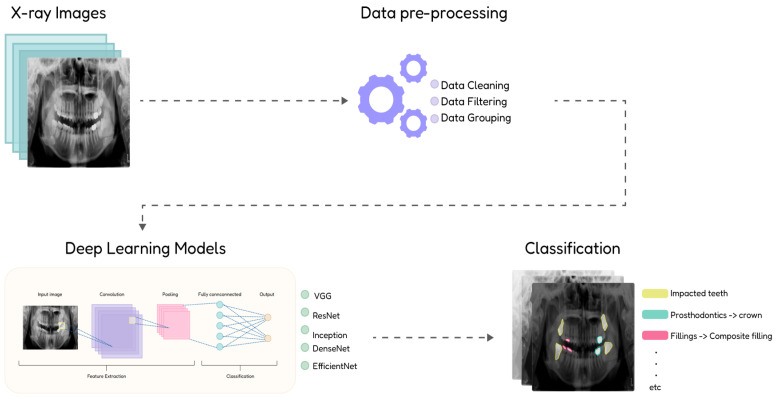
Overview of the proposed work.

This project focuses on the classification and detection of dental diseases from panoramic radiographs using advanced deep learning models. The methodology includes cleaning and preprocessing a publicly available dataset to ensure high-quality inputs for training. The performance of five state-of-the-art models—ResNet, VGG, EfficientNet, DenseNet, and Inception—will be compared to evaluate their effectiveness in identifying dental diseases. The workflow is divided into three key stages: dataset preprocessing, model selection, and comparative analysis. Figure 1 illustrates an overview of the proposed workflow, highlighting a systematic approach to achieving reliable and clinically relevant results.

### 3.1. Dataset Processing for Model Training

This subsection describes the processes involved in preparing the dataset for deep learning, including its description, types of dental problems targeted for classification, splitting, preprocessing, and augmentation strategies. All preprocessing steps and modifications were thoroughly validated under expert guidance from the Department of Orthodontics and Pediatric Dentistry at the College of Dentistry, Qassim University. This collaboration ensured alignment with established dental clinical standards and practices.

#### 3.1.1. Dataset Description

**Figure 2:**
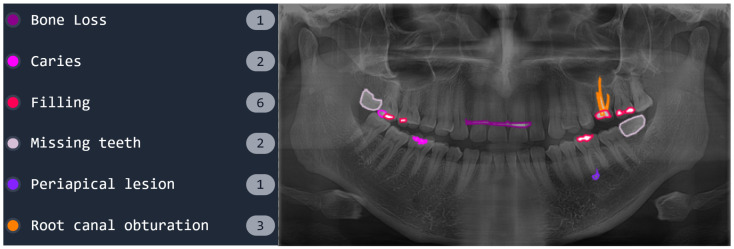
Sample panoramic radiograph of a patient with multiple dental conditions.

Panoramic radiographs provide detailed imaging of dental structures, making them suitable for automated diagnostic methods. Figure 2 shows a sample panoramic radiograph from the dataset, illustrating various dental issues, such as bone loss, caries, and impacted teeth.

**Figure 3:**
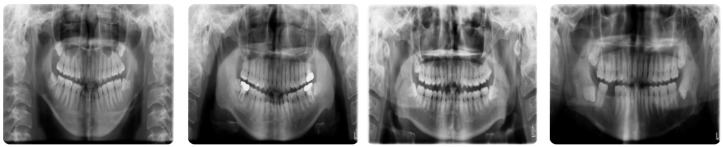
A sample of the vzrad2 Dataset [30].

For this study, we utilized the vzrad2 Dataset [30], an open-source repository of panoramic radiographs collected by Arsh’s Workspace Radio and published on Roboflow Universe, for this research. The dataset includes 10,580 high-quality images and provides 93 annotated classes representing a diversity of dental conditions. This is an extensive dataset and a good candidate for training deep learning models for dental disease classification tasks. There were, however, mislabeled data and duplicate entries and sparsely populated classes in the dataset, which required cleaning and refinement to ensure consistency and accuracy. Inconsistent labeling was corrected, and semantically equivalent terms (e.g., “Caries” and “Cavity”) were merged. Examples from the vzrad2 dataset are presented in Figure 3.

#### 3.1.2. Types of Dental Problems for Classification

The dataset is designed for the classification of various dental conditions using deep learning models. The key types of dental problems targeted include caries (cavities), which require early detection to prevent further damage; fractured and cracked teeth, which arise from trauma or excessive force and compromise the structural integrity of teeth; and periapical lesions, infections near the tooth root that require timely intervention. Other conditions include impacted teeth (e.g., blocked canines requiring surgical or orthodontic planning), bone loss and mandibular canal issues (critical for implant planning and overall dental health), and issues related to dental implants and orthodontic appliances, which require monitoring for long-term patient care.

#### 3.1.3. Dataset Splitting

The dataset was divided into 70% for training, 20% for validation, and 10% for testing. This split maximized the volume of training data while reserving adequate samples for hyperparameter tuning and final evaluation. The training set was used to fit the model, the validation set monitored performance during training, and the test set provided an independent assessment of the model’s performance.

#### 3.1.4. Preprocessing Techniques

**Figure 4:**
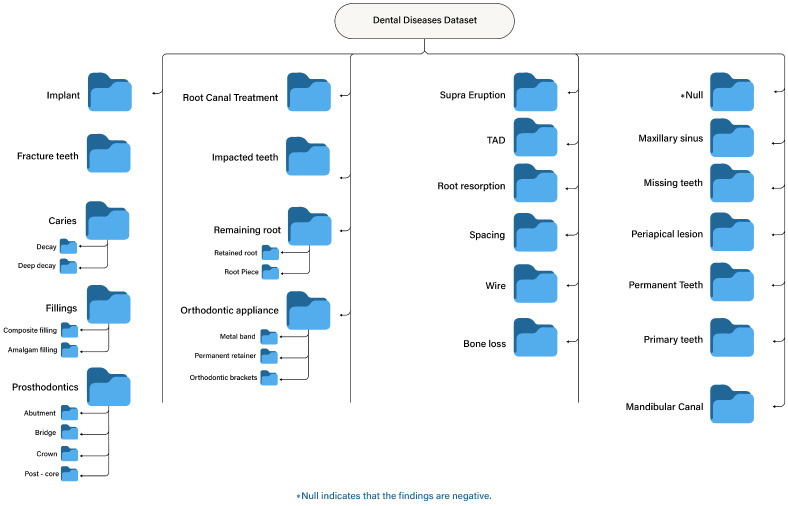
Hierarchical structure of the dental disease dataset after preprocessing.

A comprehensive preprocessing pipeline was implemented to prepare the dataset for deep learning. The first step involved handling annotations; annotations in COCO format were cleaned to remove corrupted or duplicate files. Redundant classes were removed, and mislabeling instances were corrected to ensure consistency. Class name remapping was then performed, through which semantically equivalent or misspelled terms were standardized. For example, “rct,” “Root canal obturation,” and “Root canal filling” were unified under “Root canal treatment.” Next, hierarchical merging and class consolidation were applied to group related classes under broader categories. For instance, “Bridge,” “Crown,” and “Post-core” were grouped under “Prosthodontics,” while “Composite filling” and “Amalgam filling” were merged under “Fillings”. This hierarchical structure of the dataset post-processing is illustrated in Figure 4.

**Figure 5:**
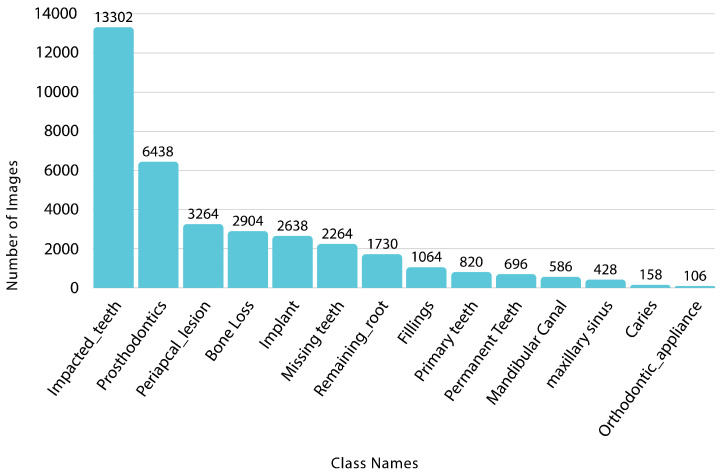
Class distribution across the dataset, illustrating the frequency of occurrences for each class.

**Figure 6:**
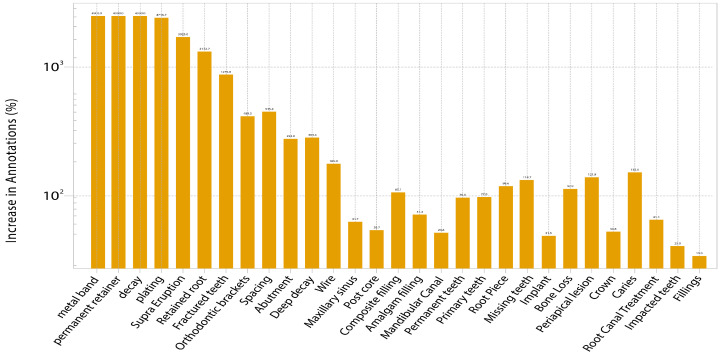
Percentage increase in sample count after class-specific augmentation (log scale).

Dataset balancing addressed class imbalance by copying underrepresented samples from validation and test splits into the training set. Rare classes (fewer than 50 samples) were augmented to ensure at least 500 instances per class. This effort reduced the imbalance ratio from 2560.4:1 to 61.4:1. Figure 5 shows the original class distribution. Meanwhile, Figure 6 illustrates the percentage increase in samples for each class, with rare conditions undergoing increases of up to 4900%, while common classes underwent more modest augmentation, effectively addressing the extreme imbalance in the original dataset.

#### 3.1.5. Data Augmentation

**Figure 7:**
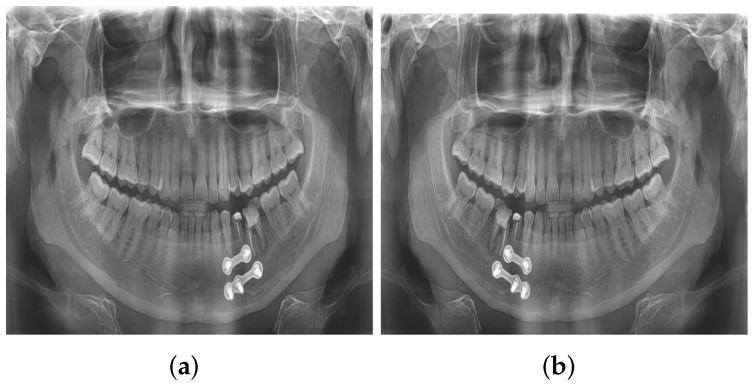
An example of flipping augmentation applied to the dataset. (**a**) Original image. (**b**) Augmented image after flipping was applied.

**Figure 8:**
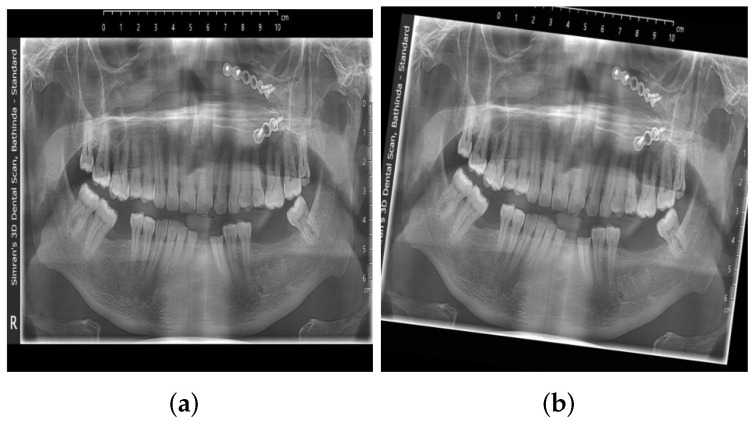
An example of rotation augmentation applied to the dataset. (**a**) Original image. (**b**) Augmented image after rotation was applied.

A class-specific augmentation strategy was implemented using the Albumentations library. For rare classes with fewer than 50 samples, augmentations included horizontal flipping (50%), brightness/contrast adjustments (±12%), rotations (up to 7°), gentle shift and scale transformations, and subtle Gaussian blur and noise. For uncommon classes (50–200 samples), augmentations included horizontal flipping (40%), brightness/contrast adjustments (±10%), and rotations (up to 5°). For moderately represented classes (200–500 samples), minimal augmentation strategies were applied to preserve the original image characteristics. Examples of augmentations are shown in Figure 7 (flipping) and Figure 8 (rotation). Overall, 3626 augmented images containing 42,811 annotations were generated.

**Figure 9:**
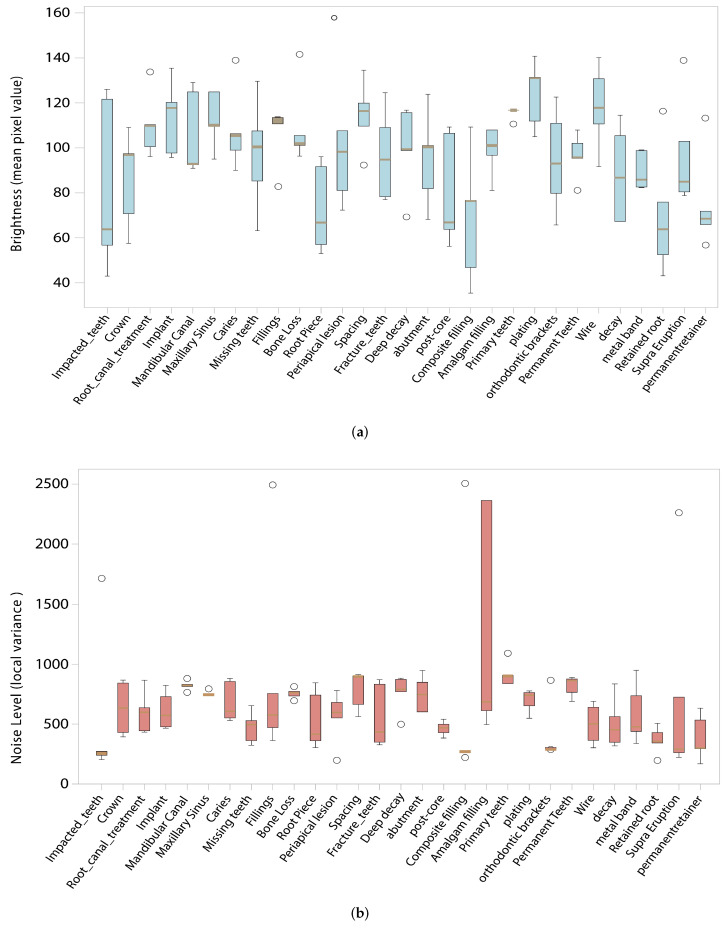
Comparison of brightness and noise levels in augmented images. (**a**) Brightness variation. (**b**) Noise levels.

Furthermore, Figure 9 demonstrates how transformation parameters were carefully calibrated for each class, maintaining clinically realistic imaging characteristics across all dental conditions.

#### 3.1.6. Avoidance of Unrealistic Radiographic Artifacts

To ensure that data augmentation did not introduce unrealistic radiographic artifacts, all transformation parameters were carefully constrained based on clinical imaging principles and empirical validation. Initial experiments using aggressive augmentation settings produced distorted images that deviated from clinically plausible panoramic radiographs, including excessive geometric deformation and unnatural intensity variations.

To address this issue, augmentation parameters were systematically refined through iterative visual inspection and validation. Rotation angles were limited to a maximum of ±7° to reflect realistic patient positioning variations. Brightness and contrast adjustments were restricted to conservative ranges (±10–12%) to preserve diagnostically relevant intensity distributions. Spatial transformations, including shifting and scaling, were constrained to small values to avoid anatomical misalignment.

Moreover, all augmented images were visually examined to determine whether important dental structures like tooth boundaries, restorations, and pathological regions were preserved. Augmentations that introduced visually implausible artifacts were excluded from the final dataset. This clinically informed augmentation strategy increased data diversity while maintaining radiographic realism, ensuring that the models learned meaningful diagnostic features, rather than artifactual patterns. The specific data augmentation techniques and their corresponding parameter ranges adopted in this study are summarized in Table 2. All augmentation operations were deliberately constrained to clinically plausible limits to ensure the preservation of anatomical realism in panoramic radiographs. These parameter ranges were selected based on empirical evaluation and visual inspection to prevent the introduction of unrealistic radiographic artifacts while improving data diversity.

### 3.2. Model Selection for Dental Disease Detection

In this study, five state-of-the-art deep learning models—VGG16, ResNet-50, EfficientNetV2, DenseNet-121, and InceptionV3—were selected for their proven effectiveness in medical image analysis tasks such as classification and feature extraction. These models have been extensively validated in prior research and are particularly suited for analyzing the complex structures in panoramic radiographs, enabling effective dental disease detection. The primary objective of this comparative approach is to identify the most effective model for the specific dataset used. These models were chosen based on their architectural principles, performance, and adaptability to medical imaging tasks. Table 3 summarizes the chosen models and their key characteristics.

The comparison in this work is intentionally limited to CNN-based architectures to enable an equitable and controlled evaluation on homogeneous training/optimization scenarios. CNNs are the most popular and clinically validated algorithms for dental radiograph analysis due to their strong inductive bias for local spatial features and their robustness on moderate-sized medical imaging datasets. Whereas new paradigms, including vision transformers and CNN–transformer hybrid models, have been shown to be effective methods for natural image analysis, their efficiency is generally limited by their requirement to operate on substantially larger datasets combined with resource demand, which can restrict their applicability in routine dental imaging scenarios. However, transformer-based architectures provide better global context modeling and long-range dependency modeling that may help reduce discrimination issues from visually overlapping dental conditions that were noted as challenging in this study. Implementing such models and investigating hybrid CNN–transformer frameworks represent a promising direction for future work.

In addition, model selection was guided by computational efficiency and deployment feasibility in clinical environments, in addition to classification performance. To provide a transparent comparison of architectural complexity and resource requirements, Table 4 summarizes the approximate parameter count and relative computational characteristics of the evaluated models.

### 3.3. Model Implementation and Training

The implementation of our research utilized the five selected models, each fine-tuned to effectively classify dental diseases from panoramic radiographs. This subsection outlines the key modifications, training protocols, and hyperparameter configurations for each model, ensuring optimal performance for the dental dataset.

While panoramic radiographs often exhibit several coexisting dental issues in the visual form, the problem in this study is a single-label multiclass classification task. As part of expert-guided class remapping and hierarchical consolidation, an image is granted one consolidated diagnostic label. Therefore, the models utilize a Softmax activation function at the final layer and categorical focal loss optimization (γ=2.0). Thus, the final classification layer for all models is made up of 35 output neurons, which correspond to the 35 consolidated dental disease classes. To model a single-label multiclass classification problem, a Softmax activation function is applied to the final layer to ensure that each panoramic radiograph is assigned to exactly one diagnostic category.

#### 3.3.1. VGG16

The VGG16 [31] model was adapted using transfer learning with pretrained ImageNet weights. To prevent overfitting, the first 20 convolutional layers were frozen, preserving low-level feature extraction capabilities. The classifier was replaced with three fully connected layers (dimensions: 4096, 4096, and the number of classes), with dropout (rate = 0.5) applied between layers. Focal loss (gamma = 2.0) was employed to address class imbalance, ensuring improved learning for underrepresented classes.

#### 3.3.2. InceptionV3

InceptionV3 [32], with its multi-scale feature extraction capability, was fine-tuned by freezing the first 7 blocks and activating the auxiliary classifier for additional gradient flow. The classifier head was adapted with a dropout layer (rate = 0.2) and a fully connected layer. The final loss combined the outputs of the main and auxiliary classifiers, weighted at 60% and 40%, respectively, to improve convergence.

#### 3.3.3. ResNet50

ResNet50’s [33] deep residual learning architecture was fine-tuned by freezing the first 6 residual blocks. A custom classifier, consisting of a dropout layer (rate = 0.2) and a fully connected layer, was added to map features to the number of classes. Focal loss was implemented to improve learning on imbalanced data. The residual connections facilitated efficient training and adaptation to dental-specific patterns.

#### 3.3.4. DenseNet121

DenseNet121 [34] was fine-tuned to leverage its dense connectivity pattern for efficient feature reuse. The first 150 parameters were frozen to preserve pretrained features, and the classifier was replaced with a linear projection and a dropout layer (rate = 0.2). This architecture naturally mitigated overfitting, enabling the effective detection of related dental conditions.

#### 3.3.5. EfficientNetV2

EfficientNetV2 [35] small was selected for its computational efficiency and accuracy. The first 30 layers were frozen, and a lightweight classifier with a dropout layer (rate = 0.2) was added. The model’s compound scaling allowed it to effectively capture multiscale features in panoramic radiographs. A gradual unfreezing of layers during training further enhanced adaptation to the dental domain.

### 3.4. Training Protocol and Hyperparameters

All models were trained using transfer learning for 15 epochs with early stopping (patience = 5 epochs) to prevent overfitting. The Adam optimizer was used with a learning rate of $1\times 10^{-4}$, while weight decay was set to $1\times 10^{-4}$ for VGG16 and $1\times 10^{-5}$ for the other models. To address class imbalance, focal loss (γ = 2.0) was employed, ensuring better learning for underrepresented categories. A ReduceLROnPlateau scheduler dynamically reduced the learning rate by a factor of 0.5 if validation accuracy plateaued for three consecutive epochs.

Data augmentation strategies, including random horizontal flipping, rotation (±10°), and brightness/contrast adjustments (±0.2), were applied to enhance model generalization. Checkpoints were saved based on the highest validation accuracy, and the best models were used for final evaluation. Performance metrics such as accuracy, precision, recall, F1-score, and mean average precision (mAP) were monitored throughout the training process. The final classification layer outputs a probability distribution over classes using Softmax activation, and training is performed using categorical focal loss to address class imbalance. The specific hyperparameters for each model, including learning rates, dropout rates, and frozen parameters, are detailed in Table 5.

In addition, all experiments in this study were performed in Google Colab, which supported a dedicated NVIDIA Tesla T4 GPU (16 GB VRAM) and about 25 GB of system RAM. The training times between runs are not guaranteed to be exact due to the shared, dynamic environment of the cloud computing environment. The training time per epoch was observed to be on the order of tens of seconds, varying by model architecture. Models such as DenseNet121 and EfficientNetV2 were lightweight, requiring shorter training times per epoch, and InceptionV3 proved the longest, due to its deeper architecture and auxiliary classifier. All reported values are averaged over multiple training runs and are included to support experimental reproducibility.

### 3.5. Model Performance Metrics

The deep learning models were assessed by widely used metrics: accuracy, precision, recall, F1-score, and mean average precision (mAP). Overall, these metrics reveal the potential and effectiveness of the models with performance accuracy, false positive (FP) prediction detection, and false negative (FN) prediction detection. The use and importance of each metric are summarized in Table 6.

By leveraging these metrics, we ensure that the results are both statistically robust and clinically meaningful, providing valuable insights into the models’ practical applicability in dental disease detection.

## 4. Results and Discussion

In this section, we compare five deep learning architectures (ResNet50, DenseNet121, VGG16, EfficientNetV2, and InceptionV3) based on the single-label multiclass classification of dental conditions. We analyze the performance of different models for diagnostic criteria to demonstrate their accuracy and efficiency in characterizing dental pathologies from panoramic radiographs, and we identify the most preferable model for clinical evaluation.

### 4.1. Learning Behavior and Model Performance Dynamics

All models were trained for 15 epochs with the same learning rate of $1\times 10^{-4}$. The curves of performance give different patterns of learning for different architectures. InceptionV3 achieved the highest validation accuracy of 97.51% at epoch 15, with a training accuracy of 98.08%, which reveals that it performed best in feature extraction and robust generalization. EfficientNetV2 performed near the edge, achieving 97.04% validation accuracy at epoch 12, and DenseNet121 achieved 96.70% validation accuracy at epoch 13. ResNet50 and VGG16 demonstrated slow growth in their performance, reaching validation accuracies of 95.45% and 95.40% at epochs 12 and 15, respectively.

**Figure 10:**
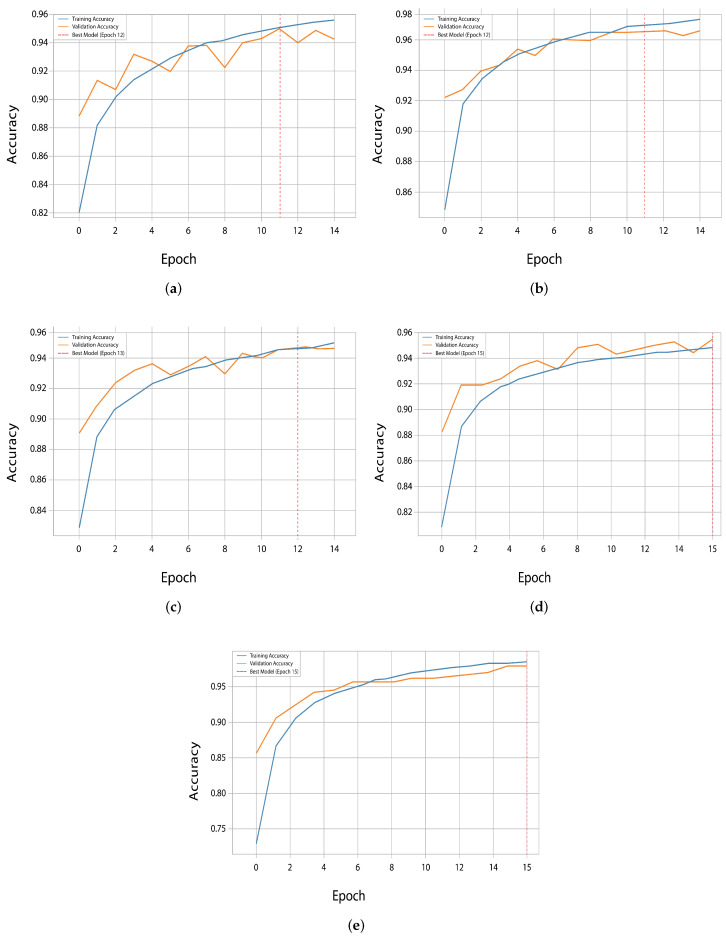
Training and validation accuracy curves for the five evaluated models, highlighting their performance trends during training. (**a**) ResNet50, (**b**) EfficientNetV2, (**c**) DenseNet121, (**d**) VGG16 and (**e**) InceptionV3.

**Figure 11:**
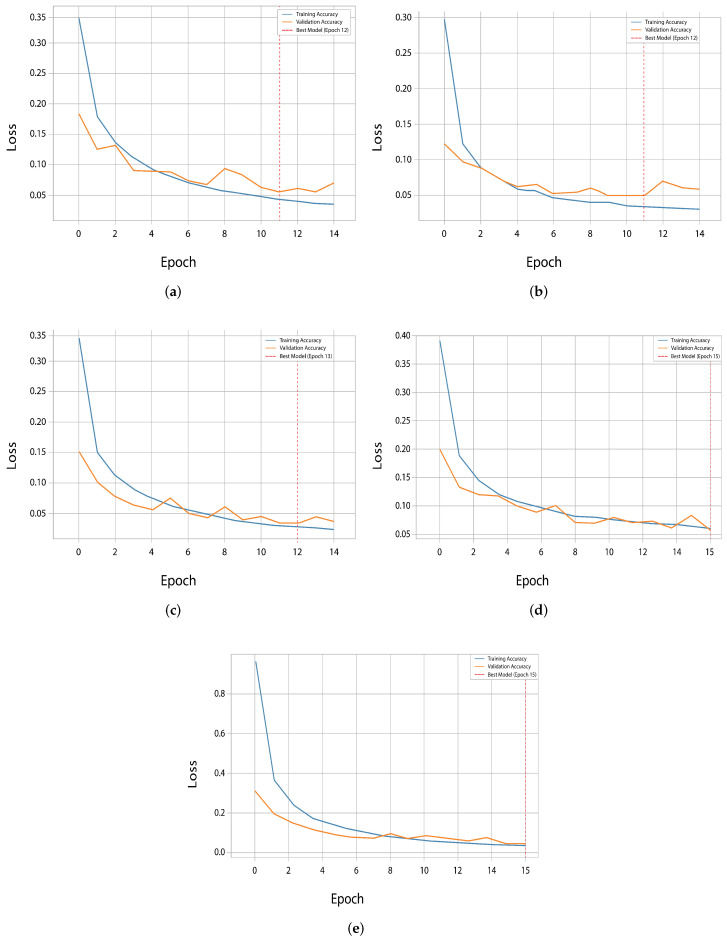
Training and validation loss curves for the five evaluated models, highlighting their performance trends during training. (**a**) ResNet50, (**b**) EfficientNetV2, (**c**) DenseNet121, (**d**) VGG16 and (**e**) InceptionV3.

Notably, EfficientNetV2 and ResNet50 were found to achieve higher validation accuracy than training accuracy during the first few epochs and thus demonstrate efficiency with transfer learning from learned weights. On the other hand, InceptionV3 showed a more normal learning curve; after the initial epochs, the training accuracy surpassed the validation accuracy fully. The loss curves provide additional details on the learning efficiency of InceptionV3, which obtained the lowest validation loss at epoch 15 and was in agreement with peak accuracy. With only limited divergence between the training and validation losses, EfficientNetV2 and DenseNet121 exhibited good generalization. However, for ResNet50 and VGG16, loss reduction was less fast and progressive. The training and validation accuracy and loss curves are illustrated in Figure 10 and Figure 11, which present red vertical lines of the epochs at which each of the models reached the highest validation accuracy.

### 4.2. Comprehensive Performance Evaluation

For a comprehensive evaluation, we assessed the models using accuracy, mean average precision (mAP), and class-wise precision, recall, and F1-scores of each model in their best-performing epochs. The performance comparison of the models is summarized in Table 7.

Within those metrics, InceptionV3 proved to be the best model with the highest accuracy (97.51%) and mAP (96.61%). Its precision (97%) and recall (96%) are a great balance, which is critical to reduce false positives and false negatives in clinical applications. EfficientNetV2 was rated second at 97.04% accuracy plus mAP of 95.79%, indicating potential as an alternative architecture. DenseNet121 obtained a total of 96.70% accuracy with mAP at 94.25% and ResNet50 and VGG16 also achieved nearly similar results with accuracies of 95.45% and 95.40%, respectively. This hierarchical performance is based on the architectural benefits of InceptionV3, which combines this multi-scale feature extraction approach with parallel convolutional filters, and is particularly efficient for complex visual structures in dental radiographs. EfficientNetV2 achieved great performance that can really be traced back to the compound scaling strategy, which balances the network depth, width, and resolution. DenseNet121 is well connected, which helped to prove its powerful learning, while ResNet50 and VGG16 are placed at the lowest tier in terms of performance, due to their simpler architectures.

**Figure 12:**
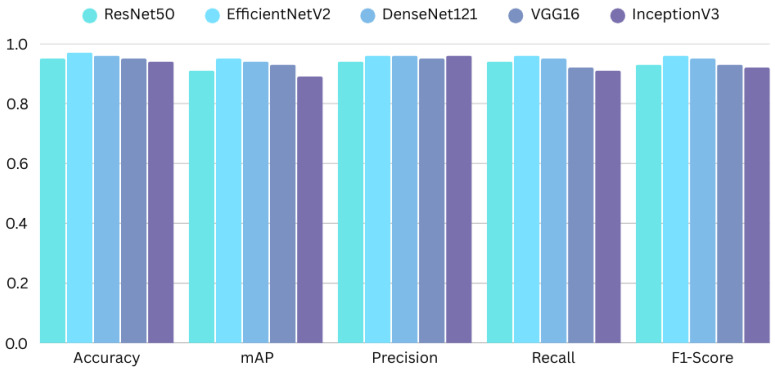
Visualization of performance metrics across all architectures.

Figure 12 presents a visualization of the performance metrics for all the architectures. We observe the highest performance for InceptionV3 in mAP and precision. This clearly signifies its capacity to make better predictions and to produce fewer false positives.

The variation in performance of the evaluated architectures is explained by the interaction of their structural design with the characteristics of panoramic radiographs. InceptionV3 had better performance than the others due to multi-scale feature extraction that can work well for panoramic images showing low contrast, anatomical overlap, and dental pathologies at different spatial scales. Parallel convolutional filters enable capturing fine-grained characteristics, including early caries and larger anatomical structures at the same time.

EfficientNetV2 also achieved high performance due to its compound scaling strategy, which preserves spatial detail while maintaining computational efficiency—an important factor for elongated and densely structured panoramic images. Due to feature reuse and better gradient flow, DenseNet121 performed better, although it was slightly less robust in visually overlapping classes. ResNet50 and VGG16 lack explicit multi-scale representations, leading to their inability to notice minute differences between pathological structures. Class-wise observations show that the majority of misclassifications were between visually similar conditions (decay and deep decay), highlighting the advantage of architectures with stronger contextual and multi-scale reasoning.

### 4.3. Impact of Class Remapping on Overfitting

**Figure 13:**
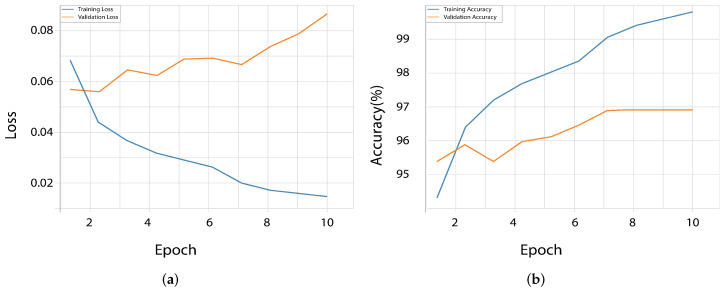
EfficientNet V2 model performance before class remapping. (**a**) Training and validation loss. (**b**) Training and validation accuracy.

During a class restructuring exercise, we looked at the effects of different methods on the performance of the models, especially for overfitting. Two methods were explored, namely, class remapping before class pre-arrangement and class organization without re-arrangement. Figure 10, Figure 11 and Figure 13 illustrate the performance of the EfficientNet V2 model under these two conditions. This finding suggests that class remapping is a major protective mechanism to mitigate overfitting. After adding class remapping, the validation and training loss curves became more aligned, and they no longer increased during the latter epochs. It indicates that this generalization of the model works better and over-fitting is reduced. We have presented in Figure 13 the performance of the model before class remapping, under which overfitting is reported, particularly related to diverging validation and training curves. By contrast, Figure 10c and Figure 11c present post-class re-mapping models in which the difference between train and validation curves is reduced, and the validation loss exhibits lower variance across epochs, reflecting better generalization and less overfitting.

### 4.4. Ablation-Style Analysis of Preprocessing and Augmentation Impact

The vzrad2 dataset involves a severe class imbalance and labeling inconsistencies that greatly influence model generalization if not mitigated well. To address these problems, a structured preprocessing pipeline that involved class remapping, hierarchical consolidation, class-specific data augmentation, and focal loss was maintained. While these steps were jointly applied in the reported experiments, their cumulative impact can be inferred from training behavior and comparative performance trends. The remapping and consolidation of classes contributed significantly to alleviating overfitting by reducing semantic redundancy and enhancing label consistency. Class remapping-based models showed an improved alignment between training and validation losses, as visualized in the learning curves (Figure 10 and Figure 11), which demonstrates improved generalization. We found this effect to be especially clear for EfficientNetV2 due to a substantial reduction in validation loss variance following remapping.

Targeted class-aware augmentation improved performance more, particularly for some of the rare underrepresented conditions, like decay and deep decay. Augmentation stabilized convergence, preserving minority-class recall without introducing artificial radiographic artifacts, while diversifying samples within clinically realistic bounds during the sample augmentation, and this resulted in an enhanced minority-class recall. These enhancements are shown alongside the high class-wise F1-scores demonstrated in Table 7. Lastly, categorical focal loss (γ = 2.0) helped achieve balanced learning, as it reduces the dominance of frequent classes in the process of optimization. This was important to achieve a significant mAP in all models, including for InceptionV3, which achieved a mAP of 96.61%. Collectively, these results indicate that efficient data engineering is the starting point in achieving the highest diagnostic accuracy. The upper performance limit comes from the architectural design, but preprocessing and augmentation strategies are critical to achieving this and fair comparisons across deep learning models. Table 8 summarizes an ablation-style comparative study of preprocessing strategies for InceptionV3, illustrating their incremental impact on performance and generalization.

### 4.5. Class-Wise Diagnostic Performance of CNN Architectures

**Figure 14:**
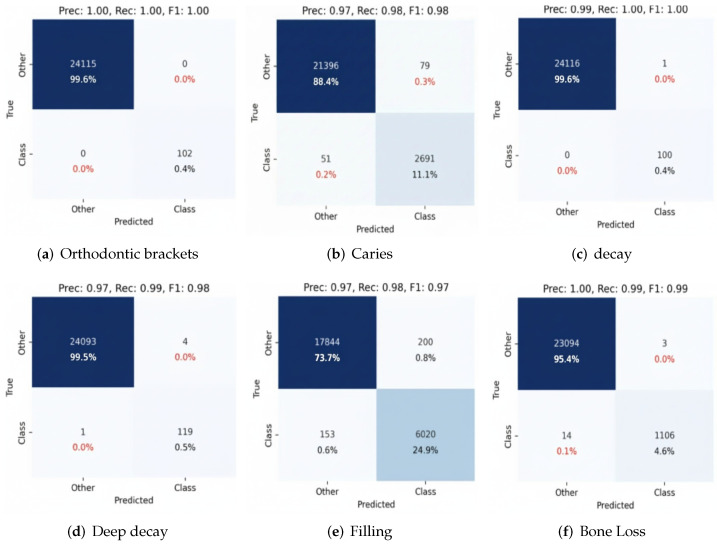
Confusion matrix for InceptionV3, showing performance across key dental conditions.

**Figure 15:**
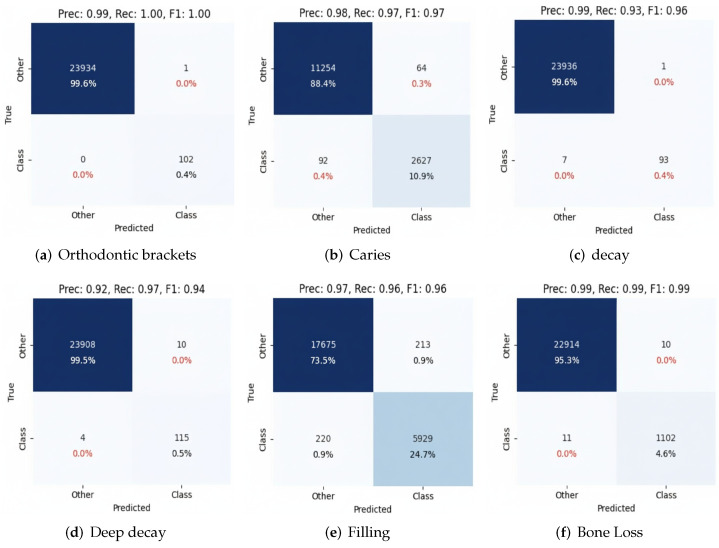
Confusion matrix for EfficientNetV2, showing performance across key dental conditions.

A performance analysis by class emphasizes the diagnostic strengths and limitations of the two best-performing CNN architectures, InceptionV3 and EfficientNetV2, for critical dental conditions, as shown in Figure 14 and Figure 15. Both models classified orthodontic brackets with almost ideal precision, recall, and F1-scores, reaching 100%, demonstrating their proficiency in classifying distinct radiographic features. Caries classification also showed strong performance, with InceptionV3 and EfficientNetV2 achieving F1-scores above 95%. However, both decay and deep decay presented greater challenges due to overlapping radiographic features. InceptionV3 and EfficientNetV2 also scored higher than previous architectures that had been tested (F1-scores = 95% and 94%, respectively), demonstrating their ability to handle complex cases effectively.

Fillings and bone loss were also classified correctly, with F1-scores above 0.95 and recall values above 0.95 for both InceptionV3 and EfficientNetV2. The results of these studies prove that InceptionV3 and EfficientNetV2 are the most stable architectures for the classification of dental conditions, owing to their advanced design features, especially multi-scale feature extraction and compound scaling, with a successful solution involving overlapping, complex feature data. From a clinical point of view, those metrics show how automated systems can diagnose simple orthodontic brackets and caries, as they can improve precision; however, further development and ensemble techniques may be needed to achieve a higher prediction for more complex conditions, such as decay and deep decay. These findings underline the high potential of deep learning models such as InceptionV3 and EfficientNetV2 to boost diagnostic accuracy and efficiency in dental radiograph analysis.

### 4.6. K-Fold Cross-Validation for InceptionV3

InceptionV3 was evaluated through a k-fold cross-validation plan to provide a uniform and unbiased evaluation of the proposed framework. The data was split into k=10 mutually exclusive folds. For each iteration, nine folds were used for training and the rest for validation. This procedure was repeated with each fold utilizing exactly once for validation in a performance evaluation across different sorts of data splits. The performance of the models was evaluated with different well-known classification tools, namely accuracy, precision, recall, F1-score, and mean average precision (mAP). A comparative summary of these metrics for all folds is presented in Table 9, providing a detailed quantitative comparison across different values of *k*.

**Figure 16:**
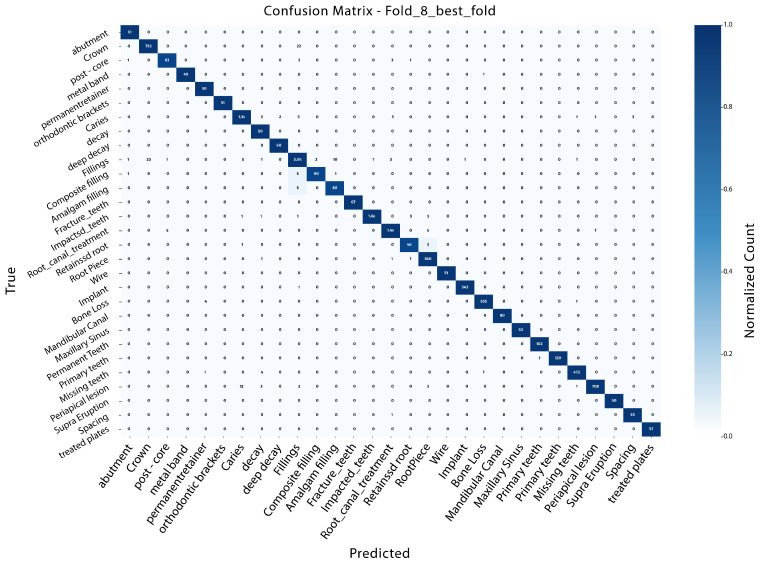
Confusion matrix of the best-performing fold (k=8) obtained using the InceptionV3 model.

The combination of these metrics yields a total score of classification correctness, robustness to class imbalance, and detection performance. Within the range of 10 folds, InceptionV3 achieves consistent, very strong and stable results. Accuracy values varied from 0.983 to 0.986, while the precision, recall and F1-score remained near 0.985 across all folds. Average performance averaged across all folds was accuracy of 0.984, precision of 0.985, recall of 0.984, and an F1-score of 0.985. The above results reflect that the model can generalize well under a range of training and validation splits. The results from all tested folds indicated that Fold 8 (k=8) demonstrated improved overall performance. This fold achieved an accuracy of 0.986, precision of 0.986, recall of 0.986, and an F1-score of 0.986, all with a mAP of 0.978. The corresponding confusion matrix for this best-performing fold is illustrated in Figure 16.

**Figure 17:**
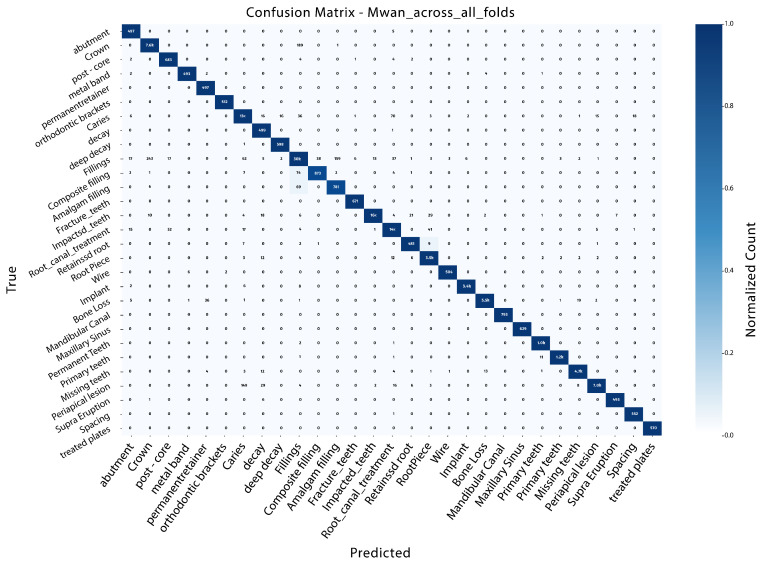
Mean confusion matrix illustrating the average class-wise performance of the improved InceptionV3 model across all cross-validation folds.

The corresponding confusion matrix clearly reflects its diagonal dominance, with high true positive percentages and low misclassification rates for most of the classes. Further on, a mean confusion matrix was generated by averaging the scores in all folds. This aggregated representation also supports the stable classification in InceptionV3, having nearly all predictions concentrated on the diagonal, which implies similar feature consistency in all categories. Figure 17 shows that the mean confusion matrix was computed by averaging the predictions across all folds.

Overall, the 10-fold cross-validation results verify that InceptionV3 is an effective and robust model in the current task. The fact that our average performance is the top-performing across all folds, along with it being comparable to the Fold 8 results, will justify its selection as the ultimate model for more experiments and analysis.

### 4.7. Qualitative Model Evaluation

**Figure 18:**
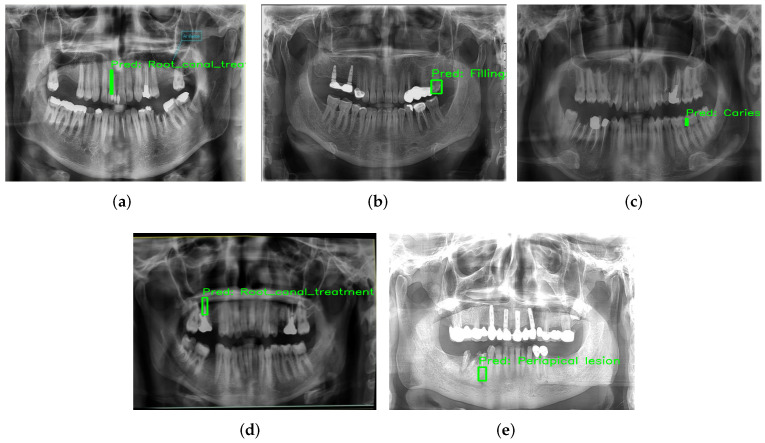
Representative qualitative results from the InceptionV3 model, showcasing the successful classification of various dental conditions. (**a**) Root canal treatment, (**b**) Filling, (**c**) Caries, (**d**) Root canal treatment and (**e**) Periapical lesion.

While quantitative metrics provide objective evidence of performance, qualitative visualization offers practical insights into the model’s clinical utility. Sample predictions from the InceptionV3 are presented in Figure 18.

Features such as periapical lesions, root canal treatment, fillings, and caries can be accurately identified and localized by the model. The predictions are very similar to annotations, showing good consistency with clinical parameters. These visual results confirm that this model is robust, as it can identify both subtle and prominent conditions in the same radiograph. Accurate spatial localization is particularly important in treatment planning, and it underscores the practical applicability of the model. In order to make the proposed deep learning model more interpretable, Gradient-Weighted Class Activation Mapping (Grad-CAM) was used to see which regions of panoramic radiographs influenced the model’s predictions most. Grad-CAM heatmaps were generated from the final convolutional layer of the InceptionV3 network for representative correctly classified test images. Grad-CAM images were produced, with typical test samples correctly predicted with a high confidence level in prediction and thus close alignment between model predictions and ground truth labels. These samples were chosen from multiple dental conditions to characterize typical diagnostic scenarios and to ensure a clinically meaningful interpretation of the model’s attention patterns.

**Figure 19:**
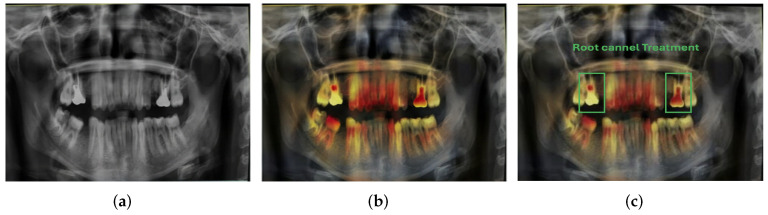
Grad-CAM visual explanation for root canal treatment classification using InceptionV3. (**a**) Original panoramic radiograph, (**b**) Grad-CAM heatmap and (**c**) clinical interpretation highlighting root canal–treated tooth.

A visual representation of Grad-CAM for root canal treatment is presented in Figure 19, confirming that the model is primarily focused on the anatomically meaningful portions of the radiopaque root canal filling materials inside the treated tooth roots. This localization is in concordance with established radiographic diagnostic criteria used by clinicians, confirming the model’s dependence on clinically relevant features, rather than spurious background factors. This pattern of attention was consistent with other dental conditions (caries, deep decay, and fillings), in which Grad-CAM maps consistently underscored lesion-specific regions (e.g., radiolucent carious areas and radiopaque restorative materials). These results support the clinical plausibility and trustworthiness of the proposed system and demonstrate its potential suitability for dental diagnostic decision support activities.

## 5. Conclusions and Future Work

This study has demonstrated the potential of advanced CNN architectures to automate the classification of dental conditions from panoramic radiographs. InceptionV3 achieved optimal performance across all scenarios. InceptionV3 was the most suitable model for clinical deployment, achieving an accuracy of 97.51% and a mean average precision (mAP) of 96.61% across the conditions that were evaluated. EfficientNetV2 and DenseNet121 also showed strong performance, while ResNet50 and VGG16 showed competitive results, revealing the overall effectiveness of transfer learning for medical imaging tasks.

While these findings indicate that deep learning–based methods are capable of significantly improving diagnostic precision and speed, this study represents a foundational step toward clinical translation, rather than a ready-to-deploy solution. The models were assessed on a single publicly available dataset, and no external validation was conducted using independent datasets from different institutions or imaging systems. Consequently, the reported performance may be influenced by dataset-specific characteristics and may not fully generalize to radiographs acquired using different scanners, protocols, or patient populations. External validation on multi-center clinical datasets is, therefore, required to confirm robustness and real-world applicability. In order to improve clinical relevance, in future steps, validation on external, multi-center data will help check the model robustness across different institutions and imaging systems, and among different patient populations.

However, the study notes that there are some limitations in the sample that arise from the many underrepresented classes and taxonomic inconsistencies in the dataset. Future work will cover more than one architecture, with specific reference to few-shot learning algorithms, explainable AI to make the information more transparent, and ensemble architectures that help leverage the complementary strengths of various architectures. Furthermore, the standardization of a benchmarking dataset and the extension of the work to other imaging modalities, such as periapical or bitewing radiographs, will expand the applicability of these systems further.

## Figures and Tables

**Table 1:** Comparative summary of dental disease classification studies.

Author (Citation)	Year	Radiography	# of Images	ML/DL Model	Validation Metrics
Kong et al. [10]	2020	Panoramic	2000	ERFNet	Accuracy = 0.89 Precision = 0.98 Recall = 0.91 F1 Score = 0.93
Mahdi et al. [11]	2020	Panoramic	1000	Faster R-CNN (ResNet-50)	mAP = 97.4% F1 Score = 96.5–97.6% Faster R-CNN (ResNet-101): mAP = 98.1% F1 Score = 97.8–98.3%
Kim et al. [12]	2020	Panoramic	303	Faster R-CNN (InceptionV3), SSD	mAP ^1^ = 96.7%
Lin et al. [13]	2021	Panoramic	895	ResNet	Accuracy = 98.33% F1 Score = 100%
Muramatsu et al. [16]	2021	Panoramic	100	CNN	Accuracy ^1^ = 98%
Cejudo et al. [20]	2021	Panoramic Bitewing Periapical Cephalometric	31,288 14,326 43,598 1176	CNN, ResNet-34, CapsNet	Accuracy ^2^ = 99.7% F1 Score = 99.1% Precision = 99.6% Sensitivity = 98.7% Specificity = 99.9%
Almalki et al. [15]	2022	Panoramic	1200	YOLOv3	Accuracy = 99.33%
Chauhan et al. [17]	2023	Periapical	428	CNN-Fuzzy System	Accuracy = 94%
Hasnain et al. [18]	2023	Panoramic	126	CNN	Accuracy = 97.87% F1 Score = 60%
Hasnain et al. [19]	2024	Panoramic	549	EfficientNet (B0–B7)	Accuracy ^3^ = 98.32% Precision = 98.32% Recall = 98.32% F1 Score = 98.37% AUC = 99.21%
Küçük et al. [24]	2025	Panoramic	407	Hybrid CNN-Transformer	mAP = 98.3% F1 Score = 96%
Zeng et al. [25]	2025	Panoramic	2467	Multi-task MMC-YOLO	–
Deo et al. [26]	2025	Histopathological	696	Ensemble Learning (ResNet50 + DenseNet201)	Accuracy = 92%

^1^ The value presented corresponds to the best-performing model (Faster R-CNN). ^2^ The values presented correspond to the best-performing model (ResNet-34). ^3^ The values presented correspond to the best-performing model (EfficientNet-B5).

**Table 2:** Data augmentation parameters and ranges used in this study.

Augmentation Type	Parameter Range	Applied Classes	Clinical Rationale
Horizontal flipping	Probability = 0.4–0.5	Underrepresented classes	Panoramic radiographs are laterally symmetric; flipping preserves anatomical validity
Rotation	±5° (common), ±7° (rare)	All augmented classes	Simulates minor patient head misalignment during image acquisition
Brightness adjustment	±10% (common), ±12% (rare)	Underrepresented classes	Reflects realistic exposure variability in radiographic imaging
Contrast adjustment	±10% (common), ±12% (rare)	Underrepresented classes	Models scanner-dependent contrast variation while preserving pathology
Image shifting	≤2% of image size	Rare and uncommon classes	Mimics small positioning changes without anatomical distortion
Scaling	0.95–1.05	Rare and uncommon classes	Simulates slight distance variation from the X-ray source
Gaussian blur	Kernel size ≤ 3	Rare classes only	Represents mild acquisition blur while preserving diagnostic details
Gaussian noise	*σ* * ≤ 0.01	Rare classes only	Simulates sensor noise without obscuring anatomical structures
Color jitter	Disabled	All classes	Avoided to preserve grayscale radiographic characteristics

* *σ* indicates the standard deviation, which is a statistical measure of variation or dispersion.

**Table 3:** Summary of deep learning models used in the study.

Model	Key Features	Advantages
VGG16 [31]	Stacked 3×3 convolutional filters, 16 layers	Simplicity, robust feature extraction
InceptionV3 [32]	Multi-scale feature extraction, factorized convolutions	Efficient feature extraction, reduced computational cost
ResNet-50 [33]	Residual connections, 50 layers	Efficient training of deep networks
DenseNet-121 [34]	Dense connectivity, feature reuse across layers	Improved gradient flow, compact architecture
EfficientNetV2 [35]	Compound scaling, Fused-MBConv layers, progressive learning	Training speed, efficiency, fine-grained feature extraction

**Table 4:** Comparison of model complexity and computational efficiency for clinical applicability.

Model	Parameters (M)	Training Time *	Inference Efficiency	Deployment Suitability
VGG16 [31]	∼138	Moderate	Low	Limited (high memory footprint)
InceptionV3 [32]	∼23.8	High	Moderate	Server-based clinical deployment
ResNet50 [33]	∼25.6	Moderate	Moderate	Standard clinical setups
DenseNet121 [34]	∼8.0	Low	High	Resource-limited environments
EfficientNetV2 [35]	∼21.5	Low	Very High	Real-time and low-resource deployment

* Relative training time refers to observed per-epoch training duration on an NVIDIA Tesla T4 GPU under identical training settings.

**Table 5:** Summary of hyperparameters configured for each deep learning model.

Model	Learning Rate	Weight Decay	Dropout Rate	Batch Size	Frozen Parameters
VGG16 [31]	$1\times 10^{-4}$	$1\times 10^{-4}$	0.5	16	First 20 layers
InceptionV3 [32]	$1\times 10^{-4}$	$1\times 10^{-5}$	0.2	16	First 7 blocks
ResNet50 [33]	$1\times 10^{-4}$	$1\times 10^{-5}$	0.2	16	First 6 blocks
DenseNet121 [34]	$1\times 10^{-4}$	$1\times 10^{-5}$	0.2	16	First 150 parameters
EfficientNetV2 [35]	$1\times 10^{-4}$	$1\times 10^{-5}$	0.2	16	First 30 parameters

**Table 6:** Evaluation metrics used to assess model performance.

Metric	Formula	Description
Accuracy	$\frac{\mathrm{TP}+\mathrm{TN}}{\mathrm{TP}+\mathrm{TN}+\mathrm{FP}+\mathrm{FN}}$	Measures the overall correctness of the model by calculating the proportion of correctly classified samples.
Precision	$\frac{\mathrm{TP}}{\mathrm{TP}+\mathrm{FP}}$	Represents the proportion of positive predictions that are actually correct. It evaluates how well the model minimizes false positives.
Recall	$\frac{\mathrm{TP}}{\mathrm{TP}+\mathrm{FN}}$	Measures the proportion of actual positive cases correctly identified, focusing on minimizing false negatives.
F1-score	$\frac{2\times \mathrm{Precision}\times \mathrm{Recall}}{\mathrm{Precision}+\mathrm{Recall}}$	Combines Precision and Recall into a single metric, calculated as their harmonic mean.
mAP	$\frac{1}{N}\sum_{i=1}^{N}AP_{i}$	Evaluates object detection models by computing the average precision across all classes, considering the area under the precision-recall curve.

**Table 7:** Performance comparison of the models based on accuracy, mAP, precision, recall, and F1-score.

Model	Accuracy	mAP	Precision	Recall	F1-Score
ResNet50	95.45%	91.29%	94%	94%	93%
EfficientNetV2	97.04%	95.79%	96%	96%	96%
DenseNet121	96.70%	94.25%	96%	95%	95%
VGG16	95.40%	93.20%	95%	92%	93%
**InceptionV3**	**97.51%**	**96.61%**	**97%**	**96%**	**96%**

Bold font indicates the best value.

**Table 8:** Ablation-style comparison of preprocessing impact on InceptionV3 performance.

Configuration	Accuracy (%)	mAP (%)	Precision (%)	Recall (%)	F1-Score (%)
Baseline (minimal preprocessing)	88–90	82–85	∼90	70–75	78–80
+ Class remapping	92–93	88–90	∼93	80–83	86–88
+ Targeted augmentation	95–96	93–95	∼95	90–92	92–94
**Full preprocessing pipeline (final model)**	**97.51 **	**96.61**	**97.00**	**96.00**	**96.00**

Bold font indicates the best value.

**Table 9:** Comparative summary of validation metrics for all *k*-values using InceptionV3.

k	Accuracy	Recall	Precision	F1-Score	F2-Score
1	0.985	0.985	0.985	0.985	0.985
2	0.985	0.985	0.985	0.985	0.985
3	0.983	0.983	0.984	0.983	0.983
4	**0.986**	**0.986**	**0.986**	**0.986**	**0.986**
5	0.983	0.983	0.983	0.983	0.983
6	0.985	0.985	0.985	0.985	0.985
7	0.984	0.984	0.985	0.984	0.984
8	**0.986**	**0.986**	**0.986**	**0.986**	**0.986**
9	0.985	0.985	0.985	0.985	0.985
10	0.984	0.984	0.984	0.984	0.984
**Mean**	0.984	0.984	0.985	0.985	0.985

Bold font indicates the best value.

## Data Availability

The dataset and code presented in this study are available at: https://github.com/its-deema/ToothTruth-Dental-Disease-Classification (accessed on 1 December 2025).
